# Recent scientific advances in leiomyoma (uterine fibroids) research facilitates better understanding and management

**DOI:** 10.12688/f1000research.6189.1

**Published:** 2015-07-06

**Authors:** Darlene K. Taylor, Kristine Holthouser, James H. Segars, Phyllis C. Leppert

**Affiliations:** 1 Department of Chemistry, North Carolina Central University, Durham, NC, 27707, USA; 2 Department of Obstetrics and Gynecology, University of Louisville, Louisville, KY, USA; 3 Department of Gynecology and Obstetrics, Johns Hopkins School of Medicine, Baltimore, MD, 21205, USA; 4 Department of Obstetrics and Gynecology, Duke University School of Medicine, Durham, NC, 27710, USA

**Keywords:** leiomyoma, uterine fibroids, uterine leiomyoma, fibroids, gynecological cancers

## Abstract

Uterine leiomyomas (fibroids) are the most prevalent medical problem of the female reproductive tract, but there are few non-surgical treatment options. Although many advances in the understanding of the molecular components of these tumors have occurred over the past five years, an effective pharmaceutical approach remains elusive. Further, there is currently no clinical method to distinguish a benign uterine leiomyoma from a malignant leiomyosarcoma prior to treatment, a pressing need given concerns about the use of the power morcellator for minimally invasive surgery. This paper reviews current studies regarding the molecular biology of uterine fibroids, discusses non-surgical approaches and suggests new cutting-edge therapeutic and diagnostic approaches.

## Introduction and context

The clinical management of uterine leiomyomas has advanced slowly and the current options remain limited. Advances in our understanding of the basic mechanisms of initiation and development over the past 5 years have elucidated the complexity of the molecular biology of leiomyomas. Although reviews of standard medical therapies have recently been published, this paper focuses on current findings in both basic and clinical research that have advanced the field and may open new strategies for treatment. Our goal is to open a dialog between clinicians and scientists to stimulate additional treatment options for women with uterine leiomyomas.

## Background

Leiomyomas, also called fibroids due to their abundant fibrotic tissue, have a 70–80% cumulative incidence in the childbearing years ^1^. These benign tumors are known to clinicians as a worldwide public health problem. Estimates of treatment costs of leiomyomas in the US range from $5.9 billion to $34.4 billion annually, and includes the costs of medical and surgical treatment, amount of work time lost and complications attributable to the tumors ^2^. These data suggest the estimated costs contribute more to health care expenditures than breast, colon or ovarian cancer ^2^. The symptoms of uterine leiomyomas include bleeding with possible subsequent severe anemia, symptoms of pain and pressure leading to difficulty with bowel and bladder function and, in some cases, infertility and pregnancy complications. Notably, the tumors may arise anywhere in the uterine myometrium necessitating individualization of therapy. Large tumors may present with few if any symptoms while small fibroids may cause severe bleeding and pain. Hysterectomy is a common treatment that unfortunately negates the possibility of childbearing. Hysterectomy aside, subserosal or intramural fibroids can negatively impact fertility ^3^. Currently, the decisive treatment is hysterectomy, either *via* abdominal/vaginal route or increasingly through laparoscopic incisions. Myomectomy, the surgical removal of only tumors, is a popular therapeutic option because it preserves childbearing, an important consideration for women of reproductive age. Uterine artery embolization, and MRI guided focused ultrasound and radiofrequency ablation are also suitable for some women.

Many advances have occurred over the past 5 years and are reviewed here briefly as they have changed our understanding of the nature of leiomyoma. Without this critical basic understanding, advances in non-invasive therapies cannot be developed and optimal individualized therapies adopted.

## Advances in basic studies

African-Americans develop benign leiomyomas at younger ages than Caucasians. The tumors appear to decrease in their growth rates before menopause in Caucasians but no decrease in growth is apparent in African-Americans ^4^. Although this finding was published in 2008, it has not always been appreciated by active clinicians and researchers. Reported similarities between leiomyomas and keloids ^5, 6^ are consistent with recent findings ^7^. Leiomyoma cells secrete high levels of disarrayed and altered collagen fibrils, fibronectin, and other extracellular matrix components and resist apoptosis ^6, 8, 9^. Fibroids vary in uterine location and size up to 20 cm, or greater. One individual may have only one tumor while another might have multiple tumors. Growth is influenced by female gonadal steroids. However, the steroid-dependent growth is tumor-specific and not systemic as the same individual uterus may present multiple fibroids having differing growth patterns—some grow, some regress, and some are stable in the same time period ^4^. Thus, leiomyomas exhibit complex mechanisms of development and growth.

Mechanotransduction, the response of cells to the mechanical forces such as compression and stretch, influences the biochemical pathways in all cells that affect growth at the cellular and tissue level ^10– 12^, including wound healing responses ^5^, growth factors, reproductive hormones and cytokines ^13^, and uterine stem cells ^14, 15^. Three recent reviews on the topic of mechanotransduction in reproduction expand in detail on this signaling mechanism ^10– 12^. Increasing evidence suggests a role for mechanotransduction in leiomyoma initiation and growth. Both biomechanical and biochemical factors, and not merely one paramount molecule, cause changes in uterine smooth muscle and leiomyoma cell behavior ^10^. These changes occur through bi-directional signaling from individual cells to their matrix microenvironment and back to fibroid cells ^10^. Catastrophic genetic alterations called chromothripsis, a sudden episode of chromosomal shattering and rearrangement, have been found in uterine fibroids ^16^. However, not all leiomyomas display these genetic alterations; thus it is not clear whether the genetic defects are a primary cause or only associated with the development of some tumors. A recent study analyzed the genetic abnormalities in 256 fibroid tumors from 120 women ^17^. In this study, 20 (7.8%) of the fibroids had a chromosomal rearrangement of 12q14-15 reflecting the rearrangement of the HMGA2 allele, while 179 (69.9%) of the fibroids exhibited a mutation of mediator complex subunit 12 (MED12), a transcription factor gene ^17^. The remaining 22.3% of the tumors were reported as having either another genetic abnormality or no detectable abnormality ^17^. Similar findings were recently found in a population of 135 women from the Southern United States with 64.33% of the fibroid tissues having MED12 mutations in exon 2 including deletion mutations ^18^. Uterine smooth muscle cells respond to mechanotransduction in a different manner from cardiac muscle, which suggests that their innate qualities are unique ^19^. One interesting aspect of leiomyomas is that they are surrounded by a relatively thick wall, a pseudocapsule, which encapsulates the tumors. Investigations of mutations in the MED12 gene have demonstrated that the pseudocapsule is derived from surrounding myometrium and not the tumor itself ^20^. Understanding of pseudocapsule development may reveal new therapeutic targets.

Interestingly, while fibroids are clonal tumors, each arising from a single cell, they are grossly and molecularly heterogeneous growths, consisting of the considerable extracellular matrix that provides the characteristic property of tumor stiffness noted on clinical palpation ^21^. Leiomyomas are rare in animals and there is no universally-accepted spontaneous animal model. The Eker rat develops tumors that resemble fibroids, but the growths do not exhibit the abundant collagen characteristic of the human tissue ^22^. While murine models have been reported, they have not been widely adopted.

Currently, research in the field relies on human tissues and cultured cells from surgical specimens, but the tumor or tumor-derived cells being studied might be in a state of active growth, or alternatively senescence at the time of acquisition. This fact is a significant consideration for the field. Because of this complexity, the identification of key molecular pathways in tumor development remains elusive and presents challenges to pharmaceutical development.

## Recent advances in clinical treatment

Clinical management decisions revolve around control of the heavy menstrual bleeding, including anemia which is often severe, chronic pain and pressure, or infertility. These symptoms are severe enough in approximately 25% of women with fibroids to require treatment ^13^. Here we review pertinent advances and suggest areas of further avenues of inquiry. Several recent articles review in detail the treatment options currently available, including herbal medications ^23– 26^, and provide clinicians with comprehensive up-to-date information for treatment decision-making. Strategies for prevention or reduction in fibroid growth rate in high-risk women may be possible, as reviewed in Table 1 ^27– 36^. It is worth mentioning that, in addition, multiple *in vivo* and animal studies suggest that Vitamin D presents an attractive strategy to prevent uterine fibroid formation ^37– 42^, and hopefully clinical trials will show the efficacy of this approach.

**Table 1.** Strategies for prevention and reduction of growth rate of uterine fibroids.

Study	Study design	n	Treatment period	Treatment regimen/dose	Main study results	Statistical significance	Summary of effects
**Green tea extract** ** (epigallocatechin gallate:EGCG)**							
Zhang *et al.* 2010 ^27^	*In vivo* Xenograft Tumors in mice	n.a.	8 weeks	Placebo (H ^2^O)	TFV 288±57	*P*<0.05	EGCG significantly reduced TFV *vs.* placebo in nude mouse model
				EGCG 1.25mg/d	129±54		
Roshdy *et al.* 2013 ^28^		33	4 months	Placebo	% TFV +24.3	*P*≤0.05	EGCG significantly reduced TFV, while TFV increased with placebo. Reduced symptom severity, improved QOL and anemia
				EGCG 800mg/d	% TFV -32.6	*P*<0.0001	
**Curcumin**							
Malik *et al.* 2009 ^29^	*In vitro*	n.a.	48 hours	No curcumin, Curcumin 5–40μM	Fibroid cell growth: All decreased	*P*<0.05	Curcumin decreased fibroid cell proliferation at all concentrations. 20μM curcumin inhibits fibroid cells, insignificant impact on matched myometrial cells *P*<0.05
Tsuiji *et al.* 2011 ^30^	*In vitro*	n.a.	3 days	DMSO (0.1%) Curcumin 100–500μM	Curcumin >200μM inhibits fibroid cell growth	*P*<0.01	Curcumin over 200μM significantly inhibited cell growth compared to control *via* PPARγ activation in fibroid cells
**Depot-** **medroxprogesterone** **acetate (DMPA)**							
Lumbiganon *et al.* 1996 ^31^	CC	910	n.a.	Controls (n=2709)	DMPA use 25.6%	OR [95% CI] 0.44 (0.36–0.55)	DMPA significantly protective against UF volume: persists >10 yrs after last dose
				Cases (n=910)	DMPA use 13.3%		
Venkatachalam *et al.* 2004 ^32^	Cohort	20	6 months	DMPA 150mg/month	TFV -33%	*P*<0.001	DMPA significantly reduces UF volume in 6 months
**Progestin releasing** **intrauterine system** **(Levonorgestrel-** **releasing** **intrauterine system:** **LNG-IUS)**							
Sayed *et al.* 2011 ^33^	RCT	59	12 months	LNG-IUS *vs.*COC	PBAC score (%) LNG-IUS=88±16.5 COC=53.5±51.2	*p*=0.02	LNG-IUS more effective than COC in reducing menstrual bleeding. Change in fibroid diameter did not occur.
					Fibroid Diameter adjusted to uterine volume (From Table 3) LNG- IUS=Baseline 2.4±1.4 Treated 2.5±1.5 COC=Baseline 2.7±1.4 Treated 2.8±1.3	*p*=0.53 after treatment	
**Combined Oral** **Contraceptive Pills** **(COC)**							
Qin *et al.* 2013 ^34^	MA	11 cohort and CC studies	n.a.	COC use	RR [95% CI]	n.a.	Meta-analysis indicates current use of COC does not increase fibroid morbidity Study heterogeneity present Conclusion: COCs do not increase risk for fibroids and thus COC should be prescribed when indicated
				Ever use (1–5 yrs)	0.88 (0.73-1.07)		
				Ever use (>5 yrs)	0.83 (0.69–0.99)		
				Current use	0.43 (0.25–0.73)		
				Former use	0.96 (0.84–1.08)		
**25-(OH) Vitamin D**							
Sabry *et al.* 2013 ^35^	CC	154	n.a.	Measured serum 25-(OH) vitamin D correlated with fibroid number and volume as determined by TVUS	Lower serum 25-(OH) vitamin D levels associated with occurrence of fibroids correlates with volume	*P*=0.01 r=0.31 *P*=0.002	Lower serum vitamin D correlates inversely with total uterine volume in black subjects, but not significant in whites.
Halder *et al.* 2014 ^36^	*In vivo* Xenograft nude mouse model and uterine fibroid cells	n.a.	Paracalcitrol 300ng/kg/day or 1, 25 di-hydroxy vitamin D 500ng/kg/ day Vehicle controls	4 weeks	∙ P-calcitrol Reduced cell growth 9% ∙ vitamin D reduced cell growth 1% ∙ Reduced fibroid size ∙ Collagen reduced in culture	*P*<0.05	Reduced tumor size, but paracalcitrol was more effective.

Abbreviations: CC, case control; COC, combined oral contraceptive pill; DMSO, dimethyl sulfoxide; DMPA, depot medroxyprogesterone acetate; EGCG, epigallocatechin gallate; LNG-IUS, levonorgestrel-releasing intrauterine system; OR, odds ratio; PBAC, Pictorial Pain, Bleeding Assessment Chart; PPARγ, peroxisome proliferator-activated receptor gamma; QOL, quality of life; RR, relative risk; TFV, Total fibroid volume; TVUS, transvaginal ultrasound; UF, uterine fibroid.

Currently, there is no simple, effective screening method to determine if a uterine tumor is indeed benign and not malignant, prior to treatment. It is known that adenomyosis can present clinically in a manner suggestive of fibroids. Recently, it was reported that experienced physicians using preoperative ultrasonograms interpreted myometrial hyperplasia on tissue histopathology as uterine fibroids ^43^. This study suggests that preoperative ultrasound imaging using current standard technology may be responsible for over diagnosing uterine fibroids. However, the misdiagnosis of leiomyosarcoma is of greater concern. A strategy to determine if a tumor is a leiomyosarcoma is urgently needed. MRI techniques demonstrate the ability to differentiate malignant from benign tumors ^44^ but have not yet been validated indistinguishing leiomyosarcoma from leiomyoma. While important, this approach is clearly not cost-effective. Using shear wave elastography, a leiomyosarcoma was accurately diagnosed pre-operatively, based on the degree of stiffness throughout the tumor ^45^. If this modality were confirmed in larger studies, it would be a major breakthrough for the field. Specifically, power morcellation has been restricted as a modality, even though it reduces complications ^46– 48^, but a reliable pre-treatment tool to diagnose leiomyosarcoma would renew interest in that method.

Ulipristal acetate was developed as a selective progesterone receptor modulator with pure progesterone receptor antagonistic activity and minimal antiglucocorticoid effects. Ulipristal is currently marketed as Esmya and was approved by the FDA in 2010 for emergency contraception. It is approved in Europe and Canada for pre-surgical treatment of fibroids. One or three month courses of ulipristal acetate has been shown to induce apoptosis and to decrease proliferation of uterine fibroid cells, and to decrease fibroid size by a variable amount. No relevant affinity for estrogen, androgen or progesterone receptors (ER, AR or PR) has been observed. Several randomized trials demonstrated that ulipristal decreased the volume of leiomyomas significantly in comparison to controls ^49– 51^. Ulipristal has also been shown to induce amenorrhea ^49– 51^. Ulipristal does not induce changes in gonadotropin releasing hormone (GnRH) levels and does not reduce serum estradiol levels below the 50 pg/dl levels necessary to maintain bone mineral density ^52, 53^. For many women the advantage of this non-surgical treatment is the ability to preserve fertility. The first study of pregnancy after completed ulipristal treatment was recently published ^53^. Of 21 women who had stopped ulipristal and attempted pregnancy, a pregnancy occurred in 15 women (71%) with 18 pregnancies during the study period with no regrowth of the leiomyomas. Twelve pregnancies produced healthy live infants and 6 resulted in spontaneous abortion (miscarriage) ^53^. Other selective progesterone modulators, such as proellex, are currently being evaluated and Elagolix, an orally administered formulation of GnRH, is currently being studied in clinical trials.

## Possible new therapies on the horizon

A purified bacterial collagenase from *Clostridium histolyticum* (CCH) has recently been shown in *ex vivo* leiomyoma tissue to significantly degrade the altered collagen when injected into tumor tissue. When the concentration of the CCH was increased and the injection volume kept small, the penetration of the CCH into the myometrium was limited and indicates that, on refinement of the dose, penetration into the myometrium could be eliminated. CCH is inhibited by serum proteins, a fact which also mitigates the concern for damage to the myometrium. Most importantly, our group in collaboration with Farshid Guilak and his colleagues at Duke University have shown that this collagenase (already FDA approved for use in the treatment of hand contractures due to collagen cord formation and for a disease of the male penis due to abnormal collagen formation), clearly reduced tissue stiffness in leiomyomas ^54^. This reduced stiffness would not only reduce the bulk of the tumor, it is theoretically capable of altering mechanical signaling pathways in the leiomyoma, overcoming the resistance to apoptosis and allowing the cells to die. Clinical studies have demonstrated that the collagenase does not affect blood vessels or nerves. The use of CCH, alone or with other drugs such as a selective progesterone receptor modulator, could potentially be utilized as an injectable therapy for uterine leiomyomas 3–7 cm in size and could be most useful in treating submucosal leiomyomas, the type most associated with infertility ^55^.

The development of materials designed to deliver and protect drug therapeutics by direct injection to the tumor site is an area of active research. Several such drug delivery materials that change phase in response to temperature changes are currently in development as they offer many advantages over conventional drug delivery systems ^23^. These thermoresponsive materials form a solution in aqueous media that reversibly transitions to a gel at physiological temperatures. The system often degrades in a defined period of time, thereby eliminating the need for surgical explantation. In its solution state, the delivery system readily mixes with therapeutic agents to afford a drug formulation that can be administered by a single injection. The injected formulation is a stable solution that transforms into a gel depot at the site of injection as a result of an elevation in temperature.

The marriage of injectable thermoresponsive delivery systems with the unmet need for viable non-surgical options for the management of uterine fibroids offers several advantages. Creating a drug depot inside the fibroid by local injection would impede diffusion and distribution of the drug away from the injected fibroid, prolong release, delay inactivation, and therefore reduce the need for repeat injections. This treatment approach for women wanting to maintain fertility yet seeking relief from fibroid symptoms could be administered by skilled individuals under ultrasound guidance in a doctor’s office. A few examples of the most promising of these thermoresponsive delivery systems are given below ( Table 2).

**Table 2.** Thermoresponsive biodegradable injectable drug delivery systems.

	AtriGel®	ReGel™	LiquoGel™
Description	Polymer (1 unit) for physically delivering entrapped pharmaceuticals	Copolymer (3 units) for physically delivering entrapped pharmaceuticals	Copolymer (4 units) for physically delivering entrapped and/or covalently linked pharmaceuticals
Properties	Water-insoluble Biodegradable Liquid: < 0°C – 30°C Gel: 37°C	Water soluble Biodegradable Liquid: < 0°C – 30°C Gel: 37°C	Water soluble Biodegradable Liquid: <4°C – 30°C Gel: 37°C
Mechanism of Drug Release	Diffusion and polymer erosion	Diffusion and polymer erosion	Diffusion and polymer erosion
Drug Release Delay	4–6 weeks	6–8 weeks	12–15 hours
Composition	Polymer platform consisting of one of the following: PLA ^1^, PLG ^2^, PLC ^3^, PAH ^4^	Triblock copolymer of: PLGA-PEG-PLGA	4 Components: Acrylic Acid Polyglycerol N-Isopropylaniline HEMA-Lac ^5^ HPG-MA ^6^
Recent applications	Testosterone reduced: Clinical studies using a depot containing 22.5 mg leuprolide maintained an effective suppression of serum testosterone (50 ng/dL) for more than 3 months.	Cancer: Single drugs were incorporated including paclitaxel, porcine growth hormone, glucagon-like peptide-1 (GLP-1), interleukin 2 (IL-2) and G-CSF	Uterine Fibroids: In Development

Notes: ^1^poly(DL-lactide); ^2^poly(DL-lactide-do-glycolide); ^3^poly(DL-lactide-co-caprolactone); ^4^polyanydrides; ^5^hydroxyethyl methacrylate-polylactide; ^6^Hyperbranched polyglycerol. Modified from reference 23

One material developed by our group and listed in Table 2 is particularly worth noting. LiquoGel™ delivers drugs similar to other thermoresponsive delivery systems but distinguishes itself from other materials in that it contains multiple functional groups that enable chemical modifications to covalently link therapeutics. Thus, multiple drugs can be delivered at one time. With the advent of the means to deliver drugs or drug combinations directly to leiomyoma tumors, the potential of reduction and perhaps eradication of tumors prior to the need for surgical or other major interventions (such as focused ultrasounds or uterine artery embolization) could be realized. Multiple drugs could be given as combination chemotherapy, such as an anti-fibrotic agent combined with a selective progesterone receptor modulator, or sequentially, for the benefit of patient care. It could be possible to deliver gene therapy in this manner as well ^55– 58^. A number of the more conventional drug therapies for uterine fibroids could be potentially entrapped or covalently linked to LiquoGel™ to afford delivery with potentially reduced side effects, improved efficacy, and controlled release profiles ^23^.

## Implications for clinical practice

Even though treatments for fibroids can be developed currently without a complete elucidation of their etiology and molecular biology, ultimately, if the molecular mechanisms for fibroid development and of myometrial proliferation are understood, additional nonsurgical therapeutic interventions may be forthcoming. Taken together, we have evidence that uterine leiomyomas grow due to cell proliferation, but even more because of excessive deposition of altered extracellular matrix due to the persistence of secreting cells. There is a growing appreciation of the complex pathways leading to the formation of uterine leiomyomas which will lead to new therapeutic approaches. Could drug therapy, either a single drug or most likely combination chemotherapy, rival the effectiveness of surgical procedures yet preserve the uterine childbearing function? If realized, could such a therapy be administered during a routine visit to the doctor or clinic? Addressing these questions presents unique opportunities at the interface of molecular medicine and clinical care.

The optimal treatment remains one that reduces the bulk of the leiomyoma and reduces blood loss while preserving the ability to have children. Clinician, doctors, patients, and researchers should continue to work together to develop cost-effective and efficacious solutions to leiomyoma disease that are compatible with the woman’s life-style, reducing or eliminating hospital stay and lengthy recovery time ^23^.
