# Supplementary material for: Recent scientific advances in leiomyoma (uterine fibroids) research facilitates better understanding and management
Source: F1000Res. 2015 Jul 6;4(F1000 Faculty Rev):183. [Version 1] doi: 10.12688/f1000research.6189.1 (PMC4513689; doi:10.12688/f1000research.6189.1)
Supplement: Peer review history

# Peer review: Referee response for version 1

**DOI:** 10.5256/f1000research.6634.r9486

**Published:** 2015-07-14

**Authors:** Pasquapina Ciarmela

**Affiliations:** 1 Polytechnic University of Marche, Ancona, Italy

I have read this submission. I believe that I have an appropriate level of expertise to confirm that it is of an acceptable scientific standard.

# Peer review: Referee response for version 1

**DOI:** 10.5256/f1000research.6634.r9356

**Published:** 2015-07-06

**Authors:** Andrea Tinelli

**Affiliations:** 1 Department of Gynecology and Obstetrics, Vito Fazzi Hospital, Lecce, Italy

I have read this submission. I believe that I have an appropriate level of expertise to confirm that it is of an acceptable scientific standard.

# Peer review: Referee response for version 1

**DOI:** 10.5256/f1000research.6634.r9355

**Published:** 2015-07-06

**Authors:** Alicia Armstrong

**Affiliations:** 1 Contraceptive Discovery and Development Branch, Eunice Kennedy Shriver National Institute of Child Health and Human Development, Bethesda, MD, USA

I have read this submission. I believe that I have an appropriate level of expertise to confirm that it is of an acceptable scientific standard.

# Peer review: Referee response for version 1

**DOI:** 10.5256/f1000research.6634.r9354

**Published:** 2015-07-06

**Authors:** Ayman Al-Hendy

**Affiliations:** 1 Department of Obstetrics and Gynecology, Georgia Regents University, Augusta, GA, 30912, USA

I have read this submission. I believe that I have an appropriate level of expertise to confirm that it is of an acceptable scientific standard.

# Peer review: Referee response for version 1

**DOI:** 10.5256/f1000research.6634.r9353

**Published:** 2015-07-06

**Authors:** Donna Day Baird

**Affiliations:** 1 Laboratory of Women’s Health, National Institute of Environmental Health Sciences, Research Triangle Park, NC, USA

I have read this submission. I believe that I have an appropriate level of expertise to confirm that it is of an acceptable scientific standard.
